# Learning to See: Using Mixed OR Methods to Model Radiology Staff Workload and Support Decision Making in CT

**DOI:** 10.1007/s42979-022-01244-4

**Published:** 2022-07-05

**Authors:** Mary Conlon, Owen Molloy

**Affiliations:** School of Computer Science, National University of Ireland, Galway, Ireland

**Keywords:** Radiology, Soft systems methodology, Discrete event simulation, Decision support, Workload

## Abstract

Demand for Computer Tomography (CT) is growing year on year and the population of Ireland is increasingly aging and ailing. Anecdotally, radiology staff reported increasing levels of workload associated with the patient profile. In this paper, we propose a framework combining discrete event simulation (DES) modeling and soft systems methodologies (SSM) for use in healthcare which captures the staff experience and metrics to evidence workload. The framework was applied in a single-scanner CT department, which completes circa 6000 examinations per year. The scanner case load consists of unscheduled work [inpatient (IP) and emergency department (ED)] and scheduled work [outpatient (OP) and general practitioner (GP)]. The three stage framework is supported by qualitative and quantitative methods and uses DES as a decision support tool. Firstly, workflow mapping and system dynamics are used to conceptualize the problem situation and instigate a preliminary data analysis. Secondly, SSM tools are used to identify components for a DES model and service improvement scenarios. Lastly, the DES model results are used to inform decision-making and identify a satisficing solution. Data from the DES model provided evidence of the differing workload (captured in staff time) for the IP and OP cohorts. For non-contrast examinations, inpatient workload is 2.5 times greater than outpatient. Average IP process delays of 11.9 min were demonstrated compared to less than 1 min for OP. The findings recommend that OP and IP diagnostic imaging be provided separately, for efficiency, workload management and infection control reasons.

## Introduction

### Background

Increasing demand for computed tomography (CT) and other diagnostic imaging services is driven by population growth and an aging population [1–3]. Worryingly, the increasing prevalence of diabetes and obesity amongst the young suggests that future elderly cohorts might suffer from a range of co-morbidities, further increasing pressure to provide diagnostic services [4, 5]. In the United kingdom demand for CTs increased by 10% and demand for MRIs by 8% in 2020, in part due to the importance of its role in many clinical pathways [1, 6].

Recently, CT played an important role in the discharge of suspected COVID-19 patients [7]. As a result of COVID-19 and the additional infection control measures required, scanner capacity has been reduced, with up to one hour of scanner downtime necessary to facilitate room decontamination and passive air exchange where scanners have been used for suspected or confirmed cases of the virus [7–9]. Previous work including an analysis of the CT waiting list found that the CT service failed to meet outpatient and general practitioner demand, resulting in a yearly increase in the CT waiting list, a problem exacerbated nationally by the COVID-19 pandemic [10, 11].

In radiology, workload has traditionally been measured in terms of the number of examinations completed [12–15]. Studies examining the workload of emergency department (ED) staff use more nuanced metrics and include patient acuteness as well as teaching, charting, answering emergency calls, reviewing diagnostic results as well as direct patient care activities [16]. Examples have been found in the literature of nursing workload metrics which include indirect and direct patient care related tasks as well as tasks unrelated to a specific patient [17]. Activity diaries have been used to capture the tasks completed by advanced practitioner radiographers and consultant radiographers and the frequency of interruptions (69.5% of reporting time was interrupted) [18]. A gap has been identified in the literature in terms of detailed measurement and understanding of radiographer workload, and calls noted for more research in this area [19, 20].

Operations Research (OR) is the art of applying analytical methods to the solution of complex management problems and has the potential to improve radiology workflows whilst incorporating staff and patient behaviors and responses [21–23]. OR provides a toolbox of methods which can be considered as either hard/quantitative or soft/qualitative. Simulation modeling is a “hard” OR problem-solving approach which allows real world problems to be described, analyzed, bottlenecks identified and alternatives considered [24]. DES, System Dynamics (SD) and Agent-Based Simulation (ABS) are the most used modeling paradigms in healthcare modeling [25].

Qualitative or soft approaches include SSM, a well-established, iterative, vehicle for action research that requires one to use the experience itself as a research object [26]. In its toolkit is Rich Picture (RP) diagramming, which allows groups to explore their information flows, communications, subconscious, occult sentiments and conflicted understandings [27, 28]. Another SSM technique is CATWOE, which focuses on creating a root definition of a service to bring forward perspectives on an issue by identifying the Customers, Actors, Transformation, Weltanschaungung or Worldview, Owner and Environmental constraints. Crowe et al. used both RP diagramming and CATWOE statements in a mixed qualitative and quantitative methods case study to acknowledge and work with multiple perspectives systematically and consider feasible and culturally desirable targeted service improvements where resources are a constraint [29].

Messy problems are those which cannot be solved with a simple, single narrow focus and require a combination of approaches [30]. Much evidence exists on the complementary nature of mixed DES and SSM approaches and the potential to facilitate stakeholder involvement in the conceptualisation, experimentation and implementation and post coding stages of OR projects [29, 31–36]. The inclusion of stakeholders offers opportunities for the internalization of knowledge, builds in-house expertise increasing the likelihood of future OR projects and increases the likelihood of implementation.

### Objective and Contributions of Paper

In this paper, a mixed methods framework is described to gain insights into the CT service by capturing metrics for staff workload and the process in terms of utilization, variation and delays. The framework has potential for application in other areas apart from radiology and is designed to educate and include those traditionally not included in problem-solving, i.e., clinical stakeholders. The paper contributes to previous literature on how hard and soft Operational Research (OR) techniques can be combined. The work contributes to the literature on radiographer workload which has previously been identified as lacking [20]. The work presents the workload experience of radiographers and healthcare assistants in a novel way, using OR methods.

## Methods

The case study hospital was a 24/7 acute surgical, medical, and critical care service with emergency and maternity services and had approximately 100 inpatient beds. In radiology, a single CT scanner provided a service for outpatient and general practitioner (OP) service work from 8.30 a.m. to 5 p.m. and a 24-h emergency service for the inpatient and emergency depart (IP) service. As identified in a previous publication, the number of unscheduled examinations being carried out (IP) is increasing yearly, while the CT waiting list for GP and outpatients is also increasing significantly (*p* = 0.014) [37].

Approval to conduct the study was obtained from the hospital’s Board of Management where the researcher was employed as a radiographer in the CT department. All data were anonymized and stored in line with local and general data protection regulatory guidelines. The identity of the radiographer as a researcher and the purpose of the research were disclosed to the staff who provided consent before interview. A pragmatic, mixed methods approach incorporating SSM was deemed appropriate given its focus on engaging multiple staff perspectives and its ability to elicit ideas for potential simulations [29, 38].

A DES model was created to capture the patient time in the system (the radiology department) and the use of staff and scanner resources during that time. It was deemed important to capture time in terms of value adding activities (being scanned, cannulated, prepared) and non-value adding (waiting for preparation, scanning, cannula removal) [39]. DES was deemed suitable due to its ability to capture discrete metrics for multiple agents and daily bottlenecks (micro) whilst also capturing the growth of the waiting list over time (macro).

**Fig. 1:**
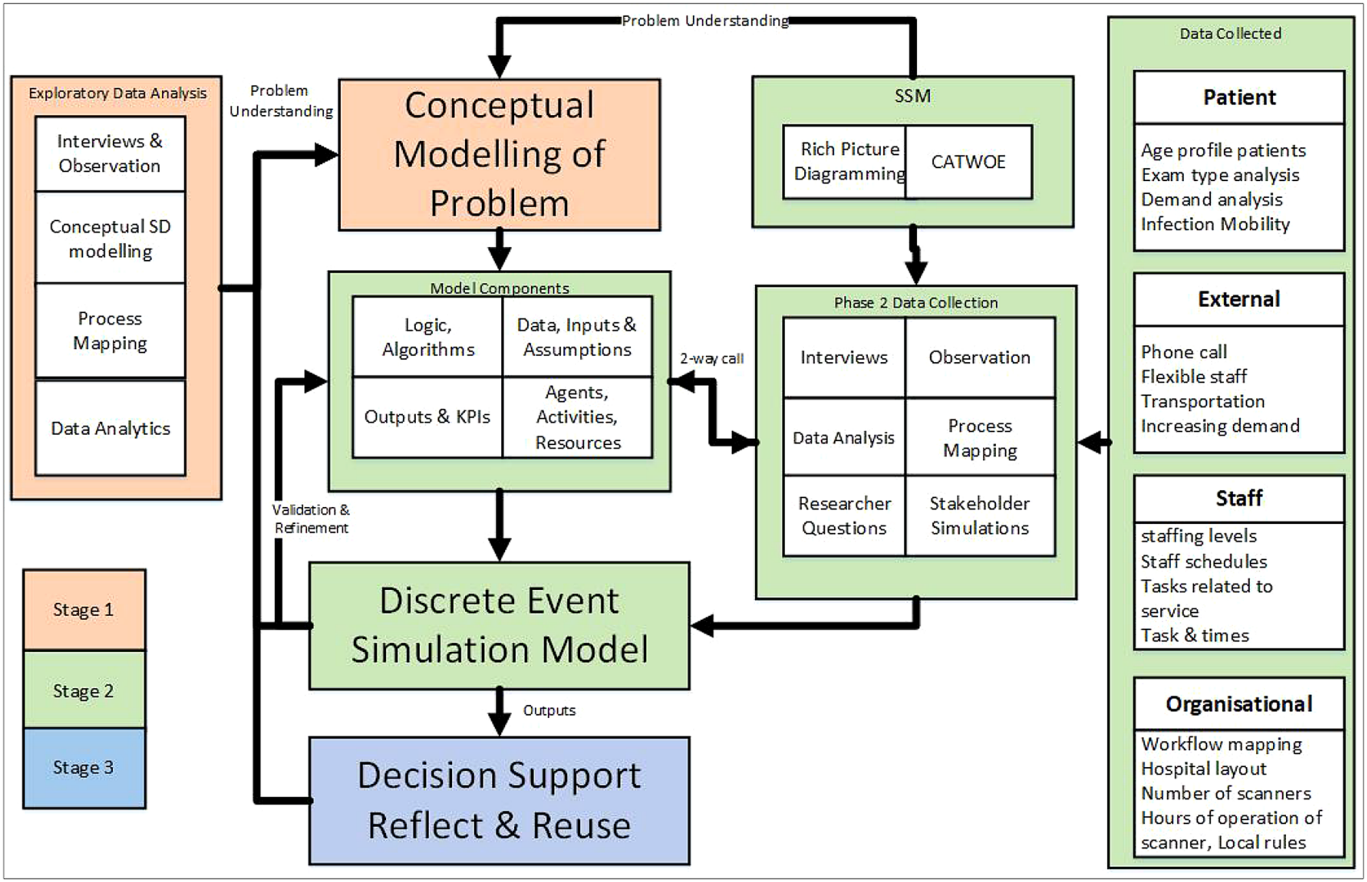
Framework components

Soft systems methodology (SSM) was used in this framework due to its strength of including varied perspectives of complex and messy problems [31, 40]. SSM was decided upon to support the initial conceptual modeling of the problem situation and to ascertain radiology staff insights. The detailed components of the developed framework are shown in Fig. 1. The exploratory stage one, employed tools such as workflow mapping, observation, interview, and system dynamics for conceptual modeling to gain an overview of the service and problem situation. Stage two adopted a SSM approach, to inform the design of a discrete-event simulation model and scenarios for testing in the final simulation model. Finally in stage three, model outputs were analyzed for each scenario, and a preferred scenario identified.

The framework allowed an iterative approach to be taken to the conceptual modeling of the problem situation where the early stages of the modeling study were revisited throughout the life cycle of the project [41, 42].

**Fig. 2:**
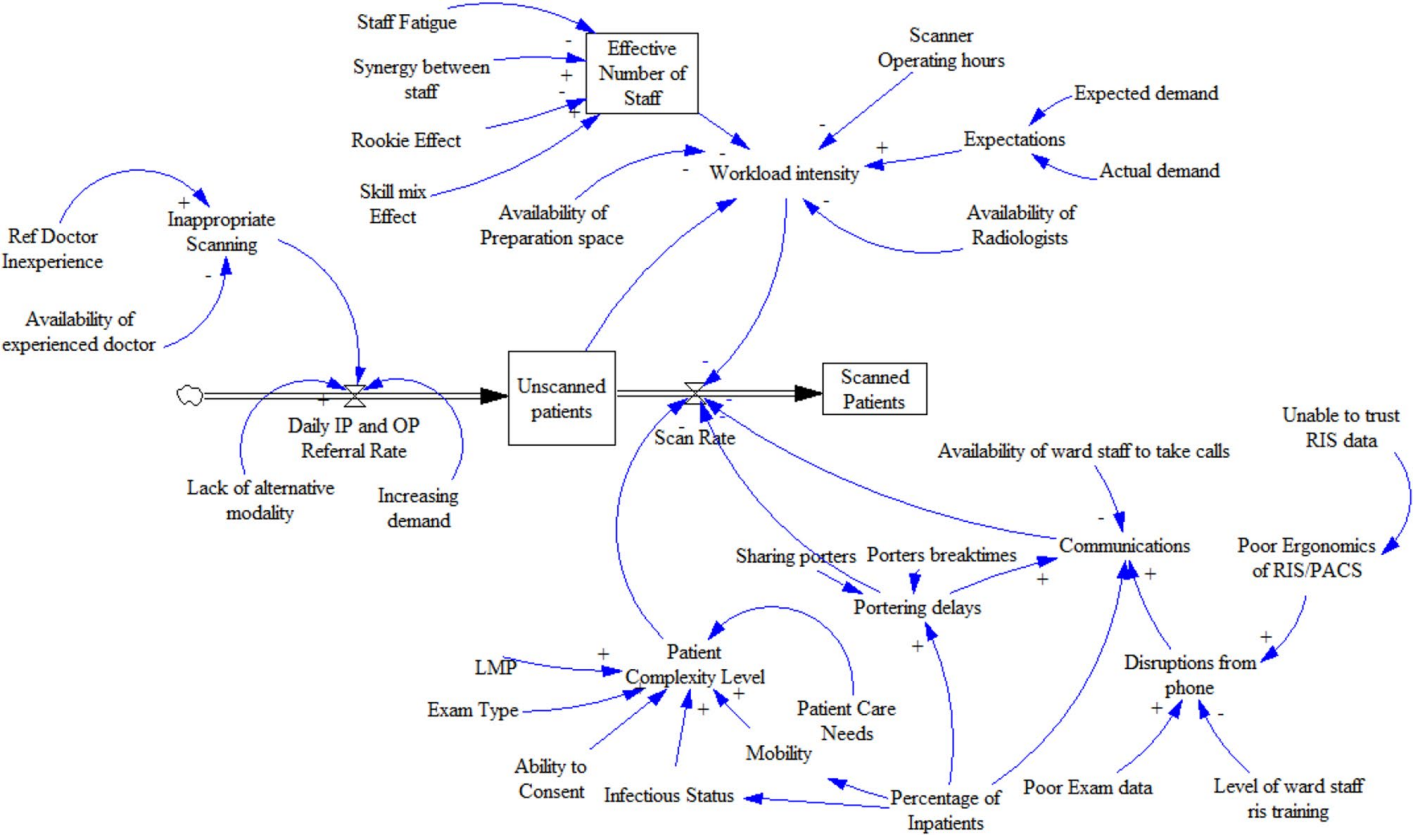
Conceptual SD model of factors affecting service delivery

Radiology staff were introduced to the concepts of mental models. Complex systems and stocks and flows diagrams during a weekly continuous professional development (CPD) meeting [43, 44]. At a later point, individual interviews with staff were held and staff were asked to contribute their input to a SD model of the factors affecting service delivery and workload. This was captured initially using pen and paper and were later formalized using the software Venism, see Fig. 2. An exploratory data analysis was also carried out to determine the waiting list growth for GP and OP and increased number of unscheduled examinations being carried out for IP and ED cases. The data analysis also included a comparison of the IP and OP populations in terms of mobility, infectiousness and examination [10].

As part of stage 2, a soft systems approach was taken to elicit the salient factors affecting service delivery and identify components for inclusion in a DES model of the service. Again, a staff CPD meeting was used as an opportunity to introduce radiology staff to SSM and the potential to create shared understandings of problem situations using free hand drawings [40, 45–47]. Interviews were conducted to elicit from staff the important aspects of the service by identifying the customers, actions, transformation process, worldview, owners and environmental constraints (CATWOE) of the CT service [29, 42]. Convenience sampling was used to recruit interview participants (*n* = 5), which included a CT clinical specialist, a department manager, ED doctor, a porter and ED nurse. Staff were interviewed individually in their place of work and notes were taken on a pre-printed document with sections for each part of the CATWOE mnemonic.

The CT clinical specialist, two senior CT radiographers, and one radiologist were directly involved in the RP session. The facilitator was a member of staff from the radiology department. During a picturing session, participants were presented with a blank page and a set of colored markers, and were asked to draw freehand graphics representing their interpretation of the service [27]. Participants were instructed to avoid the use of text where possible [28, 48]. Questions were encouraged throughout the session and the facilitator/radiographer researcher prompted throughout so as to uncover difficult to observe workflows and communications. Where text could not be avoided comments and speech bubbles were written directly onto the drawing and a list of perceived issues was generated. While not directly involved in the RP session, the worldview and environmental constraints of the porter, ED doctor and ED nurse obtained from the structured interviews were added to the RP by the facilitator.

To create a version of the RP which could be disseminated, formal drawings were created to represent the hand drawings of staff. Handwritten text was typed and added to the softcopy version. Once completed the RP was presented to staff members for final discussion and refinement [28].

**Fig. 3:**
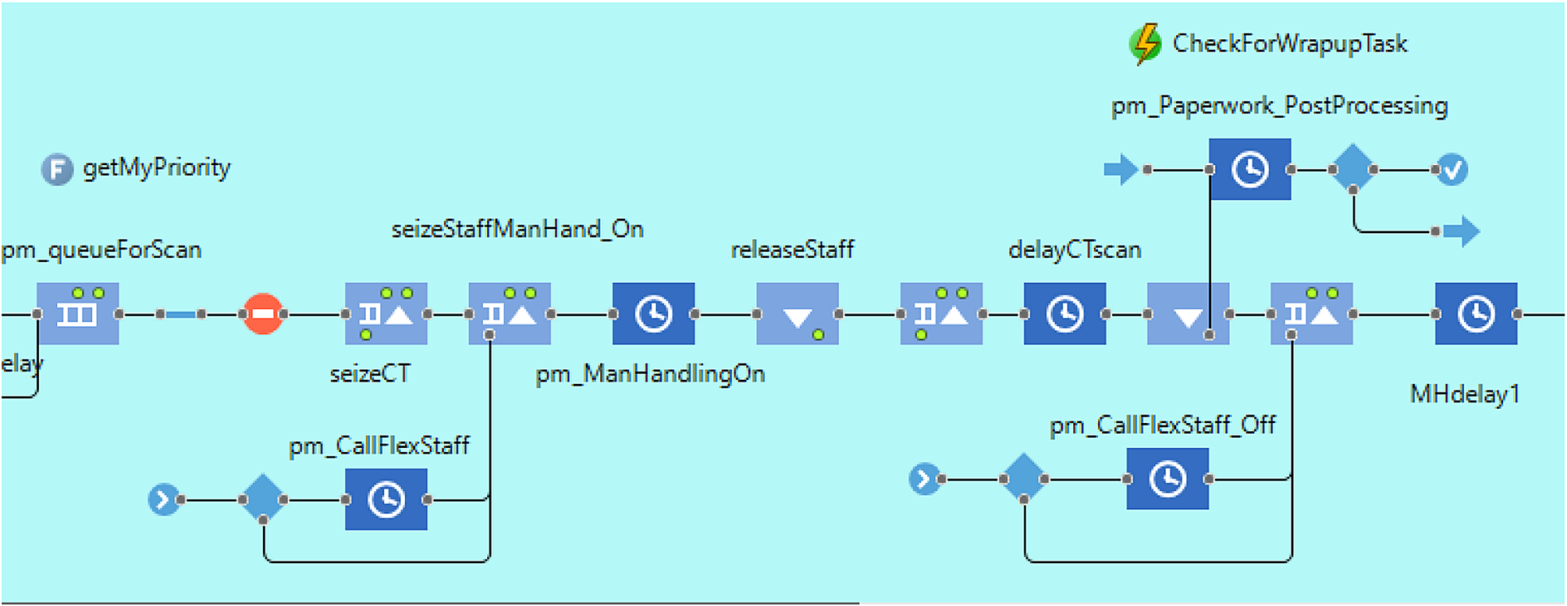
Patient preparation section of DES model

Using the software AnyLogic (University Edition 8.4) a DES model for the CT department and service was created. Model parameters were informed by stages 1 and 2, as was logical process identified in the workflow diagrams. Activities such as patient preparation, transportation, manual handling, infection control measures, scanning, cannula insertion and removal, image post processing and paperwork were captured in the DES model (see Fig. 3 for patient scanning section of model). Data were obtained from various information systems and task time parameters were determined through observation and verified with the CT clinical specialist. A discussion with radiology decision makers (Radiology manger and Clinical Director) was arranged to agree scenarios for testing in the DES model.

### Model Validation

A combination of techniques was used to validate and verify the model’s accuracy:

1. Statistical validation against historic data for the patient time in the system
2. Statistical validation against historic data for the waiting list evolution.
3. Face-to-face validation with radiology clinical stakeholders.

**Fig. 4:**
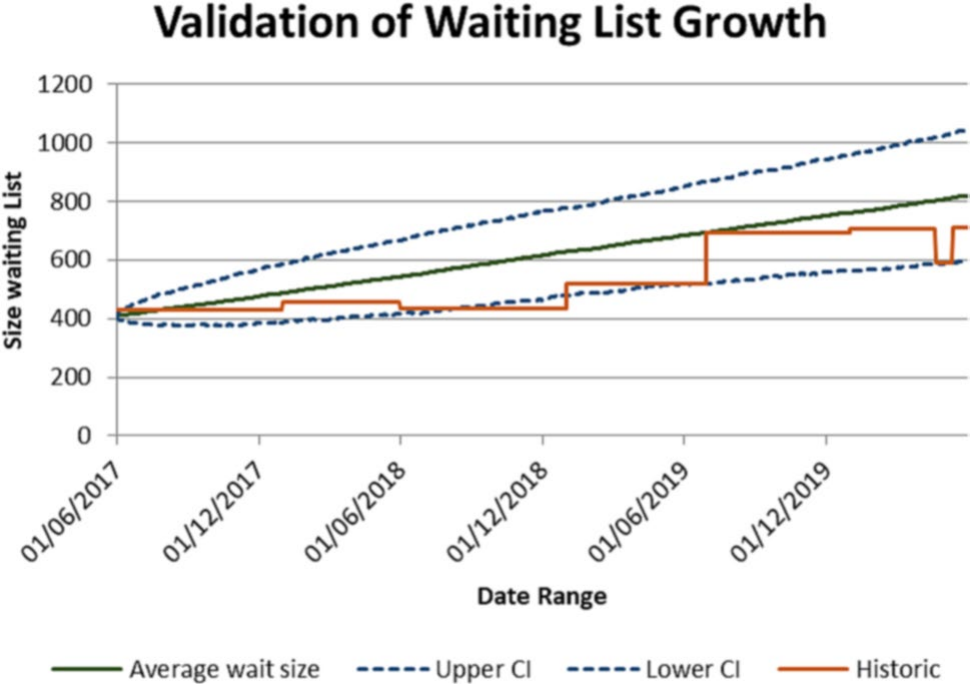
Simulated and historic growth of waiting list over 3 years

The model was validated, using the patient time in radiology from arrival to scan finish, for all examinations (*n* = 5958) for 365 days based on arrival times from historical data from 2015. Overall a mean error difference of 3.36 min between the modeled process times and the historical process times was calculated. The predicted waiting list evolution is shown in Fig. 4 which includes the historical waiting list evolution over the period.

Model validation and verification ensured that the model sufficiently captured the current mixed IP/OP service for the intended purpose of gaining insights and support decision-making in regards to the problem of CT waiting lists.

## Results

### Qualitative OR Results

**Fig. 5:**
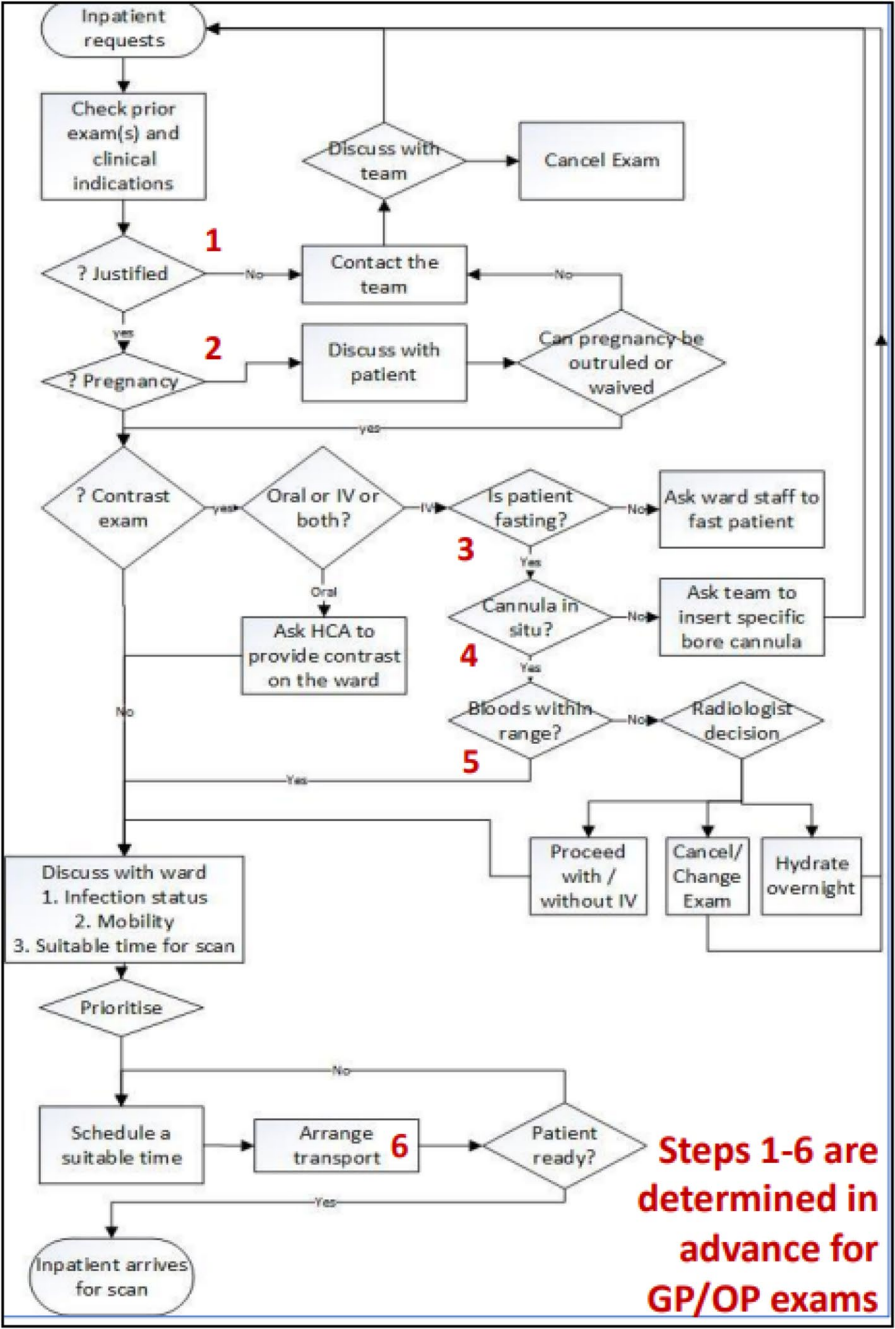
Outpatient versus inpatient scheduling steps

Stage 1 of the framework resulted in the production of a conceptual model jointly created by decision makers and clinical staff, using system dynamics notation (Fig. 2). The exercise was used to inform the exploratory data analysis and to reinforce learning from an earlier presentation on system dynamics. The subsequent exploratory analysis highlighted the differences between the IP and OP cohorts in terms of mobility, infectiousness and exam type referrals, thus providing model parameters for each patient cohort sources as well as new insights for staff [10]. Workflow mapping provided evidence of the complexity of IP scheduling compared to OP scheduling. IP scheduling usually occurs on the same day as scanning whereas the scheduling of OP cases is completed by clerical staff weeks or months in advance (see Fig. 5). The figure demonstrates 6 steps for IP cases that must be completed by radiographers on the day of scanning, which can be completed in advance for OP cases.

It is noted that many questions (denoted by diamond shape) must be answered before a CT exam can be safely scheduled and completed. In the case of scheduled exams these questions can be pre-empted, with answers documented in advance of the patient arrival. Conversely, for unscheduled examinations the verification and collection of information required occurs immediately prior to the examination being completed. During the model building and analysis stage, some changes to workflow were made, such as the method of scheduling a porter. Workflow mapping instigated a workflow change where HCA staff arranged transportation when preparing patients instead of radiographers having to call for patients. As is often reported, the process of workflow mapping resulted in a better understanding of and improvement of the system [49–51]. The difficulty created for the modeler was that process maps became obsolete and a decision needed to be made whether to model the original or changed system. While an inconvenience for those carrying out the research these changes provide evidence of action research, as unimposed changes resulted from the very process of researching the problem [47, 52, 53]. Gaining buy-in for the project was aided by ensuring that important and relevant issues were identified, and that the work resulted in quick tangible changes and benefits to the department [54].

Rich Picture diagramming and CATWOE were used to elicit insights from staff and decision makers. Table 1 provides details of a CATWOE statement and root definition for the CT service drawn up from the perspective of the radiographers providing the service. It describes the purposeful activities of the staff providing the service and the transformation process which occurs.

**Table 1:** CATWOE statement and root definition for CT service

Customers Patients who require a CT scan or interventional procedure and referring doctors who require a diagnostic report and images for their patients. Patients may be from the OP department, IP wards, AMAU, ED or referred from their dentist, physiotherapist or GPs
Actors Radiographers scan patients under the direction of the radiologists on behalf of referring doctors, assisted by HCA, nursing staff, clerical staff, porters
Transformation process Patients are scanned and cared for. Referring doctors are provided with diagnostic images and/or a report. The referring doctor’s questions are answered
Worldview or Weltanschauung We want to meet the needs of the patients by providing them with a diagnostic report and a safe service. We want to meet the needs of referring doctors in a timely manner to contribute to the patient’s management
Owners Head of department, RSM, Hospital management. Competent authorities for patient protection in relation to medical exposure to ionizing radiation
Environmental constraints All CT examinations must be justified and radiation dose kept as low as reasonably achievable, patient safety, consent and care must be ensured. There is only 1 scanner providing a full service from 8.30 a.m. to 5 p.m. with a 1 h lunch break Monday to Friday. An emergency service is provided 24 h a day, 7 days a week. Not all radiographers are CT trained or able to cannulate patients on commencement of work. Patient priority can change and the needs of the most urgent cases must be met first. The HSE has national time frame within which to scan patients
Root definition A safe radiology service delivered to consenting patients of varying urgency and from various sources for justified examinations, to facilitate referring doctor who make decisions based on the findings from high-quality diagnostic images and reports

**Fig. 6:**
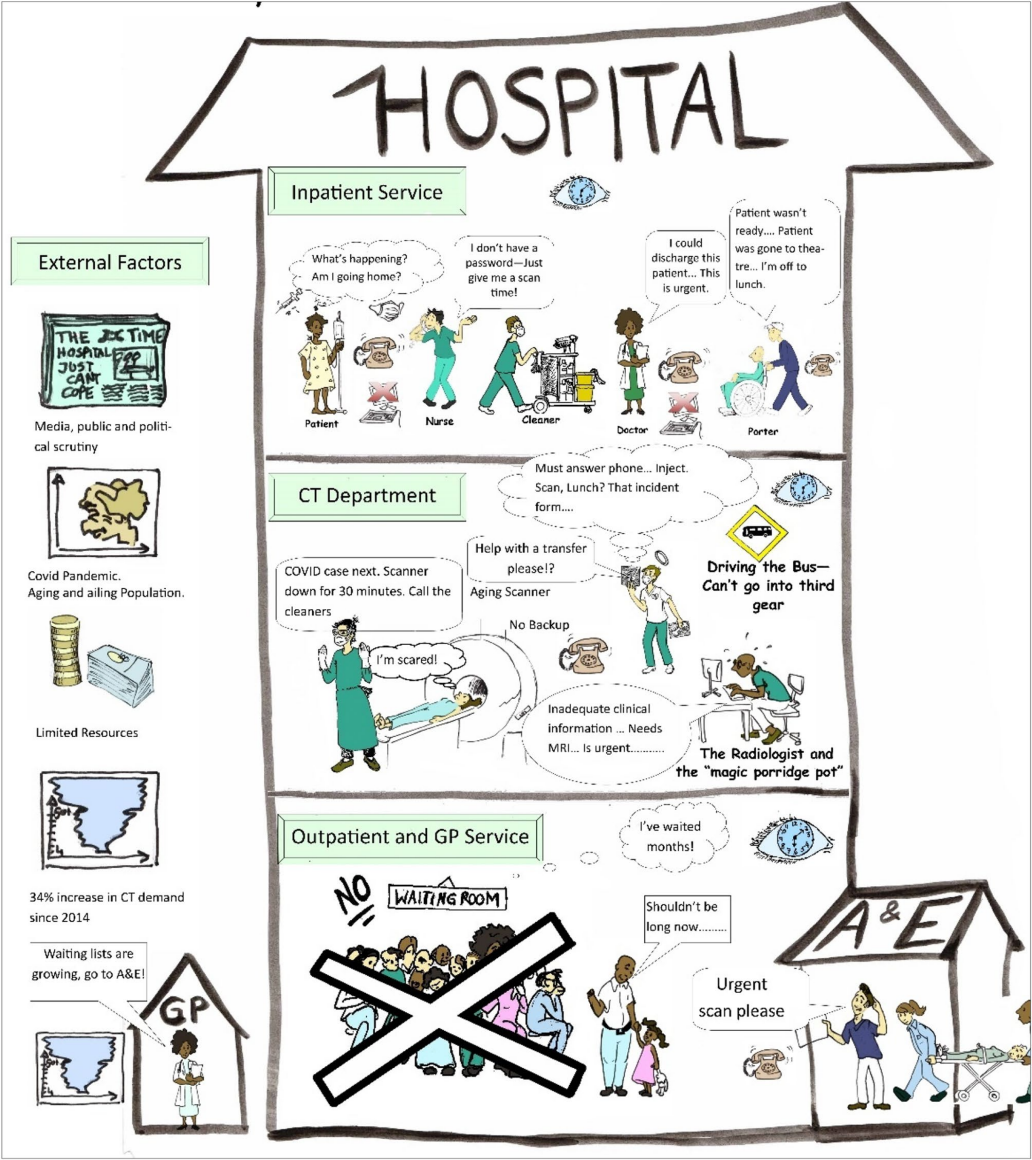
Rich Picture diagram created for the CT service

Figure 6 shows the final augmented Rich Picture and captures the key features of the CT service, such as staff activities, the process, the environment, the delays, distractions and external factors contributing to workload and affecting service delivery. A clock and phone are visible in each room to represent the time sensitive nature of the work and constant “often repetitive” communications occurring between staff. Background chatter whilst scanning is a common occurrence. A clear definition is made between the inpatient and outpatient services by placing these groups on separate floors of the hospital. The inpatient service includes the acute medical assessment unit and accident and emergency department, whose patients generally require an immediate service. The GP in the community is depicted and their awareness of growing waiting lists. GP and outpatient waiting lists appear as an external factor as these do not impact the daily operations of the service and are only a concern at management/decision-maker level.

A graphic representing an inpatient depicts how patient complexity varies in terms of a patient’s care needs, infectiousness, mobility and exam complexity. The outpatients are seen to be experiencing delays and the staff are conscious of the inconvenience a delay causes and feel empathy.

The frustration of the staff nurse as they seek to confirm a patient’s future scan time is also depicted. They just want a verbal answer and do not want to refer to the information system; they may not remember their password or may imagine a phone call is quicker than logging on to the RIS/PACS. Bad habits have appeared over the years and they are conditioned to expect verbal confirmation of a time. They are under pressure to ensure a scan happens in a timely manner because they know discharge is dependent on it or are aware the patient is waiting a long time or is deteriorating.

External factors affecting service provision are grouped to the left of the diagram and appear outside of the drawing of the hospital. It was agreed to locate waiting lists as external as they were not a concern for CT service provision on a daily basis. Age and infection and chronic diseases and newspaper headline also appear externally. A total of 32 issues and constraints were documented during the RP diagramming sessions and through interviews with a sample of these provided in Table 2, (see previous publication [37]).

**Table 2:** Sample of issues identified by varied staff members

Source	Perceived issue
Clinical specialist	Overall demand is increasing and the CT service has multiple referral sources with patients of varying priority, priority may change over time. Constant reprioritization is required Phone calls and visits from the various referral sources cause time delays and distract radiographers who are scanning. Staff want verbal confirmation of scan times even though this information is available on the RIS
Radiographer1	In order to have all the information I need to hand: I have to transcribe information onto a paper schedule. Some use the RIS but this works for me and saves me from going in and out of multiple screens, multiple times or relying on my memory Delays occur when staff are not available for the manual transfer of patients from their bed/trolley to the CT scanner and back again
Radiographer2	The skill mix amongst the radiographers and percentage of staff able to cannulate and inject patients has been depleted due to recent staffing changes Delays result where transportation is not immediately available for inpatients, this may be due to porter or wheelchair shortages or where patients on the wards are not ready to leave the ward when the patient arrives
Radiographer3	Quite often someone forgets to arrange transportation for the inpatients who are drinking on the wards. There can be up to 3 calls per inpatient to arrange preparation and transportation and to discuss whatever time’s been allocated to them We need a dedicated workstation for planning—there are constant demands for the PC from multiple staff which breaks concentration when planning. The Lab system and RIS/PACS systems should be side by side or on the same PC
Porter	At break times we may only have one porter covering several areas Patients are not always ready to be transported when we arrive on the ward and we have to ring back to CT to explain, or we think they are going to need a wheelchair but we arrive and they need a bed

### Quantitative OR Results

**Fig. 7:**
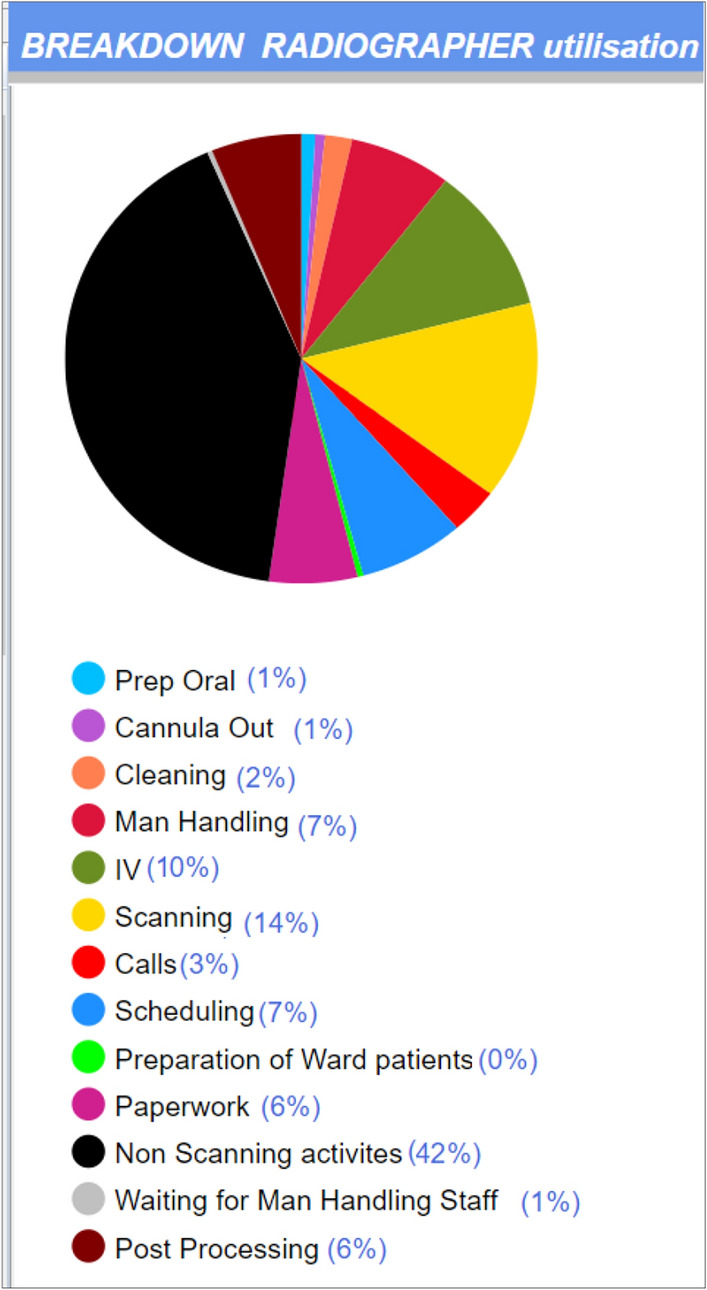
Radiographer utilization captured using DES modeling

The outputs from the DES model pertained to the staff, patients, and the process. Pie charts, created in AnyLogic, were used to present a breakdown of staff activities for weekdays for radiographers and healthcare assistants including administrative, clinical and non-clinical tasks associated with scanning Fig. 7. Average staff utilization rates of 58% and 38% were found for radiographers and HCA, respectively.

Table 3 provides workload related metrics, created by clinical stakeholders, for the IP and OP cohorts (scheduled and unscheduled). Metrics for staff and scanner utilization, as well as number of tasks completed were also obtained from the model.

**Table 3:** Metrics captured in the model

IP/OP comparison metrics	IP	OP	Explanation of result
Average perturbations	11.9 min	0.15 min	Perturbations are delays to process attributed to patient type. Seen to be greater for IPs
Consumed staff minutes (CSM) for IV and Oral exams	47.05 min	36.5 min	The staff time consumed for exam preparation, scanning and manual handling, observation for IV exams. Greater for IPs by 22%
Consumed staff minutes for non-contrast	16.5 min	6.2 min	The staff time consumed for exam preparation, scanning and manual handling, observation for non-contrast exams is 2.5 times greater for IPs
Percentage of time scanning (scanning time/CSM) for non-contrast exams	38.91%	61.85%	For OPs 22.94% more of radiographer time is spent scanning for non-contrast exams

## Discussion

### Learning to See

The framework (Fig. 1) allowed for the involvement of clinical staff and decision makers throughout all three stages providing them with a formal introduction to SSM and SD and DES modeling. Staff who attended the CPD sessions on each topic were provided with certificates as evidence of participation for their portfolios. Some recognized benefits of involving staff and decision makers in the project included, providing them with an opportunity to internalize research knowledge, promotion of trust and consensus building and a more meaningful focus, higher likelihood of implementation and educating decision makers to know how and where modeling can be useful [50, 54–56].

The use of the mnemonic CATWOE offered staff a means to reflect on the service they worked in and resulted in the capturing of a root definition of the CT service, Table 1. Staff acknowledged they had not previously considered the transformation process which occurs for their patients or their own worldviews and appreciated being asked what constraints they worked under.

Figure 5 demonstrates 6 steps for IP cases that must be completed by radiographers on the day of scanning. These can be completed in advance for OP cases, reducing the cognitive workload for these prescheduled exams. Coupled with this, the exploratory data analysis also identified that IP are less mobile and more likely to be positive for infection [10], inferring higher workload for IP. The RP diagram (Fig. 6) and SD model (Fig. 2) alluded to the complexity of IP scheduling, and the importance of the task to the smooth running of the CT department. Workflow mapping (Fig. 5) best demonstrated this complexity and the non-technical skill set required to schedule IP exams [57]. It is recommended that formal training using a decision support flow chart such as Fig. 5, be given to staff responsible for scheduling. Where feasible, the “planning” radiographer role should be differentiated from the more technical and physical patient scanning and patient preparation roles, through perhaps the allocation of a separate workspace to avoid interruptions, information loss and errors [16, 58–61].

DES captured detailed metrics not previously available to managers on the breakdown of radiographer and healthcare assistant activities (Fig. 7), affording them an opportunity to consider how staff add value to the process [62, 63]. Staff expressed concern at these apparently “low” rates of utilization captured by the DES model (58% for radiographers and 38% for healthcare assistants); however, two points must be made. First, the result represents involvement in physical activities and does not include the time spent on cognitive tasks such as scheduling (see Fig. 5). The second point is that there was no baseline with which to compare this metric and there is no reason to consider this as underutilisation of staff resources. A radiation oncology center used DES to model their process as part of quality improvement initiative and reported similar utilization rate of 56% for staff and 58% for equipment [64]. It should be noted that no baseline for diagnostic radiographer utilization, capturing 13 tasks in total, has previously been captured using DES and that the baseline applies to the case study hospital and its mix of Inpatients and Outpatients, its workflow and CT demand. A comparison across radiology sites would require the same means of modeling to be used for both.

The SSM tools allowed the modeling project to incorporate knowledge elicited from a variety of stakeholders. Through the RP diagram, the case study offered rich insights into work conditions that contribute to stress levels and cognitive workload including physical conditions, time pressures associate with the work, the significant role of relationships and how work is organized [14]. A by-product of the SSM approach was the list of issues, which represent opportunities to improve departmental performance and quality of the service, see Table 2. This list of issues provides staff with an opportunity to voice their issues and to appreciate the perspective of others such as portering staff.

A disadvantage of the use of RP diagramming was its subjective nature and difficulty of reproduction. Additionally, the method of recording was note taking which means some data may have been excluded and post hoc scrutiny is not possible. While recognizing that the approach yielded subjective insights rather than testable results, the advantages of participant involvement included the potential to identify a greater variety of scenarios and process metrics [21, 26, 28, 50]. The final RP was constructed using the software Microsoft paint and Microsoft publisher contravening the predominantly freeform and unstructured nature favored for RP generation [27]. As the purpose of the model was to convey a shared understanding and to disseminate to a wider community a more professional finish was required. The RP does not purport to describe every CT department’s service claiming instead to be an interesting representation of the reality of those interviewed [26].

For non-contrast examinations, IP were found to consume 2.5 times more staff resources (captured as staff time) than OP. The DES model also captured delays caused by individual patients, with IP responsible for on average 15 min delay compared to less than 1 min for OP. Delays occur where it is necessary to find additional staff for manual handling or where the scanner is unavailable for infection control reasons (cleaning and drying) Table 3. A recommendation was made to provide separate IP and OP services.

Extrapolating the case study findings to a national level, we recommend the establishment of regional diagnostic hubs to provide a dedicated scheduled service for the outpatient and general practitioner CT examinations. A scheduled service would benefit from increased efficiency, reduced variability, and a reduction in downtime due to infection control measures. From the patients perspective, vulnerable inpatients would benefit from not sharing waiting areas and corridors with outpatients and those who accompany them, improving their experience of privacy and dignity [65].

This recommendation comes with the caveat that greater access to CT should be considered as a partial solution to the problem of CT waiting lists but not as a solution to the increasing demand for diagnostics. By increasing patient access and meeting demand for CT services, the supplier induced demand effect could result in an increased use of ionizing radiation per capita [66]. It is seldom sufficient to change one aspect of a problem and see substantial improvement in a system [30]. A combination of policies will be required to maintain or improve quality and should include balanced strategies that include preventive programs as well as care and treatment [67].

## Framework Limitations and Future Work

In medicine, lacunae are small spaces, cavities or gaps occurring in bone. In OR, lacunae are gaps where deep fundamental questions remain unanswered. Ormerod asked of modeling projects whether the right people were involved and whether the voice of the affected but uninvolved was heard [68]. A limitation of this framework is that the voice of the affected patient was unheard, and patients were not involved in the identification of process metrics and RP diagramming. In hindsight, an assumption was made that the clinical staff represented the patient’s needs. Pearson et al. contend that in a democratic society, it should be unthinkable that service re-design takes place without the involvement of the people most directly affected, arguing that processes such as modeling that are used to inform the decision-making process should involve patients and members of the public [69]. Therefore, a greater element of PPI (Patient and Public Involvement in Research) is advised for future work.

A wider study including referring doctors such as GP and OP doctors is recommended. Perhaps, it might be discovered that they require more from radiology than improved access to examinations, such as the facility to discuss what examinations are appropriate [70]. A widening of the scope of the RPD and involvement of additional stakeholders at a conceptual level has the potential to identify new levers for change, ensuring we do not solve the wrong problems or create new ones [71].

## Conclusion

This framework allowed the insights and experience of multiple staff members and decision makers to be jointly considered**,** connecting the modeler, clinical staff, and decision makers from problem conceptualisation to decision support. The objective of this work was to create an OR model which would capture metrics related to staff workload and also to support routine decision-making by predicting waiting list evolution under differing scenarios. This work recommends that shared regional “outpatient only” scanners be used to provide community scanning for work which can be scheduled in advance. Such a solution would allow grouping of similar cases to reduce set-up times and increase throughput and in doing so allow greater Outpatient and GP access to diagnostics.

The framework with its integration of qualitative and quantitative OR techniques helped to generate findings of low and high level, which were of direct practical relevance to decision makers in radiology. Most importantly, in-house decision-making abilities were also improved through the education of staff on the concept of healthcare system dynamics and operating within constraints. Staff were challenged to imagine how their system could perform better and interact better with other systems such as the A&E department or the GP in the community. This is fitting considering that the key challenges facing healthcare providers in future years may be more organizational and logistical in nature than medical and scientific [72].

## Data Availability

Model available upon request.

## Code availability

Model available upon request.
